# [Retracted] MicroRNA‑195‑5p inhibits the progression of hemangioma via targeting SKI

**DOI:** 10.3892/etm.2024.12767

**Published:** 2024-11-18

**Authors:** Bin Sun, Zhi Huang, Hua Yang, Xuya Zhao

Exp Ther Med 23:165, 2022; DOI: 10.3892/etm.2021.11088

Following the publication of this paper, it was drawn to the Editor’s attention by a concerned reader that certain of the Transwell cell invasion assay data shown in Fig. 2E on p. 4 and Fig. 5C on p. 6 were strikingly similar to data appearing in different form in other articles written by different authors at different research institutes that had already been published elsewhere prior to the submission of this paper to *Experimental and Therapeutic Medicine* (one of which has already been retracted).

In view of the fact that the abovementioned data had already apparently been published previously, the Editor of *Experimental and Therapeutic Medicine* has decided that this paper should be retracted from the Journal. The authors were asked for an explanation to account for these concerns, but the Editorial Office did not receive a reply. The Editor apologizes to the readership for any inconvenience caused.

